# Characterization of a novel MSH2 variant in Lynch syndrome: clinical data and complementary bioinformatics assessment

**DOI:** 10.31744/einstein_journal/2026AO0757

**Published:** 2025-12-01

**Authors:** Anisse Marques Chami, Thalia Rodrigues de Souza Zózimo, Carolina Guimarães Ramos Matosinho, Agnaldo Lopes da Silva, Maria Raquel Santos Carvalho, Letícia da Conceição Braga

**Affiliations:** 1 Postgraduate Program in Obstetrics and Gynecology Faculdade de Medicina Universidade Estadual Paulista “Júlio de Mesquita Filho” Botucatu SP Brazil Postgraduate Program in Obstetrics and Gynecology, Faculdade de Medicina, Universidade Estadual Paulista “Júlio de Mesquita Filho”, Botucatu, SP, Brazil.; 2 Department of Genetics, Ecology and Evolution Universidade Federal de Minas Gerais Belo Horizonte MG Brazil Department of Genetics, Ecology and Evolution, Universidade Federal de Minas Gerais, Belo Horizonte, MG, Brazil.; 3 Department of Gynecology and Obstetrics Universidade Federal de Minas Gerais Belo Horizonte MG Brazil Department of Gynecology and Obstetrics, Universidade Federal de Minas Gerais, Belo Horizonte, MG, Brazil.; 4 Postgraduate Programa in Genetics Universidade Federal de Minas Gerais Belo Horizonte MG Brazil Postgraduate Programa in Genetics, Universidade Federal de Minas Gerais, Belo Horizonte, MG, Brazil.; 5 Laboratório de Pesquisa Translacional em Oncologia Instituto de Ensino, Pesquisa e Inovação Instituto Mário Penna Belo Horizonte MG Brazil Laboratório de Pesquisa Translacional em Oncologia, Instituto de Ensino, Pesquisa e Inovação, Instituto Mário Penna, Belo Horizonte, MG, Brazil.

**Keywords:** Lynch syndrome, Neoplastic syndromes, Hereditary, Colorectal neoplasms, DNA mismatch repair, MutS homolog 2 protein

## Abstract

**Objective:**

To describe the clinical characteristics and perform a multi-step bioinformatics evaluation of the pathogenicity of NM_000251.3(MSH2):c.1894_1898del (p.Ile633Lysfs*9), an MSH2 germline variant detected in a family with Lynch syndrome.

**Methods:**

Clinical evaluation included description of phenotype, family history, and immunohistochemical characterization of the proband’s tumors. For pathogenicity classification according to the American College of Genetics and Genomics/Association for Molecular Pathology (ACMG/APA) criteria, bioinformatics analyses included: (i) literature and database screening, searching for the variant allele frequency, case reports, or functional studies, including ClinVar, VarSome, Ensembl, PubMed, EVA, and ABraOM; (ii) prediction of variant impacts using ExPASy Translate, Pfam, and Modeller 9.24; and, (iii) mechanisms that could mitigate the effects of the variant included alternative splicing and exon skipping (UniProt and GTex) and nonsense-mediated decay (NMD; MutationTaster2021).

**Results:**

The proband, a 55-year-old female, was diagnosed with two metachronous colorectal cancers. Immunohistochemical analysis showed loss of expression ( *MSH2* in one tumor, and MSH2 and MSH6 in the other). Seven deceased family members were diagnosed with cancer (four colorectal, one uterine, and two unspecified). This variant caused a stop codon in MSH2 exon 12 of 16. When translated, the protein loses 294 C-terminal residues, which may prompt protein degradation. If the mutated protein escapes degradation, dimerization and DNA-binding domains will be present. Therefore, negative dominance effects were possible. No isoforms ending in exon 12 have been identified in the literature or in RNA splicing databases. A stop codon before the last exon-exon boundary indicated the occurrence of NMD.

**Conclusion:**

No evidence of protein-rescuing mechanisms was found, supporting the classification of this variant as likely pathogenic/pathogenic.

## Highlights

Family identified with recurrent colorectal cancer, suggestive of Lynch syndrome.

A new variant was detected, NM_000251.3(MSH2):c.1894 _1898del (p.Ile633Lysfs*9).

The variant causes a frameshift mutation leading to a premature stop codon, suggesting pathogenicity.

Using bioinformatics tools, we investigated possible escape mechanisms, such as the presence of isoforms without the mutated exon.Location of the variant suggests that it causes non-sense mediated decay, which is reinforced by immunohistochemistry findings showing absence of MSH2 protein expression.

Detailed assessment allows this variant to be classified as probably pathogenic/pathogenic.

Data from the article was extracted from the thesis by author Anisse Marques Chami presented to the *Faculdade de Medicina* of the *Universidade Estadual Paulista “Júlio de Mesquita Filho* ”, Botucatu Campus, to obtain the title of Doctor from the Graduate Program in Obstetrics and Gynecology, Concentration Area: Oncology, in 2022.

## In Brief

Familial cancer is a complex condition requiring genetic counseling. Screening for genetic variants is necessary in addition to clinical diagnosis. An identified genetic variant must be classified according to its potential pathogenicity. We identified a novel variant in the MSH2 gene, in a family with clinical manifestations compatible with Lynch Syndrome. Bioinformatics analysis showed that the variant created a frameshift mutation, leading to a premature stop codon. No compensatory mechanism was identified, confirming that this variant as likely pathogenic.

## Graphical abstract

**Figure:**
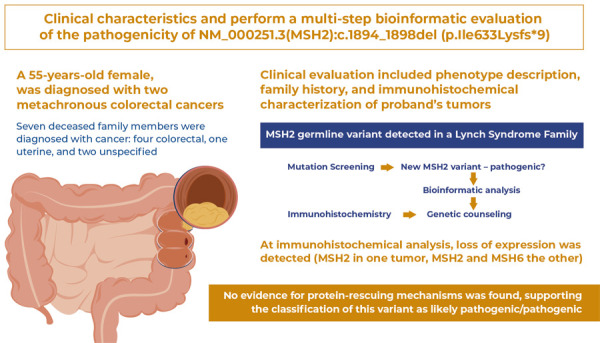

## INTRODUCTION

Lynch syndrome (LS; OMIM 120435) is amongst the most common hereditary cancer predispositions.^( 1 - 3 )^It includes gastrointestinal, gynecological, skin, urinary tract, and central nervous system cancers.^( 2 )^ Colorectal and endometrial cancers are the most frequent in LS, and it has been estimated that 3% of all colorectal cancers and 3% of endometrial cancers are associated with LS.^( 4 , 5 )^ Lynch syndrome is an autosomal dominant condition with incomplete penetrance and heterogeneous clinical manifestations caused by deleterious germline variants in the post-replicative mismatch repair (MMR) genes *MLH1* (OMIM 120436) *, MSH2* (OMIM 609309) *, MSH6* (OMIM 600678) *,* and *PMS2* (OMIM 600259). In addition, germline *EPCAM* microdeletions or epimutations can influence *MSH2* expression and cause LS.^( 2 )^ Cancer risk varies according to the mutated genes. Pathogenic variants in *MLH1* and *MSH2* present higher penetrance, with the risk of colorectal cancer exceeding 50%, whereas *PMS2* and *MSH6* pathogenic variants present lower penetrance.^( 6 )^ Among other functions, MMR proteins correct DNA replication errors, reduce mutation rates, control the fidelity of meiotic recombination, block recombination between non-homologous sequences, and help deal with environmental mutagens by mediating DNA damage-induced apoptosis.^( 7 - 9 )^

In recent decades, knowledge of the genotype-phenotype correlations for each LS gene has allowed the development of risk reduction management protocols for patients with pathogenic variants in MMR genes.^( 6 )^ Complete anamnesis (including family history), tumor pathology, microsatellite instability analysis, and immunohistochemistry of MMR proteins are crucial for guiding the diagnosis and management of LS. The Revised Bethesda Guidelines and Amsterdam (I and II) clinical criteria enable the identification of high-risk patients or families.^( 10 - 12 )^ Prediction models, such as the PREdiction Model for gene Mutations (PREMM5), also help screen individuals who may be mutation carriers.^( 13 )^ Furthermore, the inclusion of immunohistochemistry and/or microsatellite instability analysis in Universal Testing for all colorectal and endometrial cancers is currently recommended to improve the identification rate of individuals at risk of LS.^( 14 )^ However, as LS is defined by the presence of a pathogenic variant in one of the MMR genes,^( 2 )^ genetic-molecular studies are fundamental for identifying germline variants and establishing their phenotypic impacts.

Several mutations in MMR genes have already been described in LS and are shared in the main international variants databases, such as InSiGHT, VarSome, and Human Genetic Variation Database (HGVD).^( 15 - 17 )^ The description of mutations and associated phenotypes assists in the pursuit of better care by identifying specific population or family risk groups.^( 14 )^ It is fundamental to accurately characterize the pathogenic potential of newly identified genetic variants, and the strategies required vary according to each variant. Recently, we identified a new variant, NM_000251.3(MSH2):c.1894_1898del (p.Ile633Lysfs*9), in a Brazilian family with LS. In VarSome, this variant was classified as pathogenic according to the American College of Medical Genetics and Genomics and the Association for Molecular Pathology (ACMG-AMP) criteria.^( 18 )^ Nonetheless, no clinical description has been associated with this variant and no investigation has been conducted thus far, to evaluate the mechanisms underlying its pathogenicity.

## OBJECTIVE

In this study, we aimed to describe the phenotype associated with NM_000251.3(MSH2):c.1894_1898del (p.Ile633Lysfs*9) and present a step-by-step bioinformatics analysis for characterization of its pathogenetic mechanisms.

## METHODS

The proband was referred for genetic counseling, considering LS as a diagnostic hypothesis. The proband was a 55-year-old female, diagnosed with metachronous colorectal cancer in the previous year. The first tumor was in the sigmoid colon and the second was diagnosed 12 months later, in the cecum. Both tumors were pathologically described as moderately differentiated invasive adenocarcinomas. The anatomopathological records did not describe mucinous, signet ring cell histologic features, medullary growth patterns, or infiltrating lymphocytes in the tumor. Immunohistochemical analysis of MMR proteins showed loss of MSH2 and MSH6 expression in the first tumor, and loss of MSH2 expression only in the second tumor. At cancer staging, both tumors were classified as initial, without signs of invasion or spread. Consequently, both tumors were resected, and no further oncological treatment was required.

**Figure 1:**
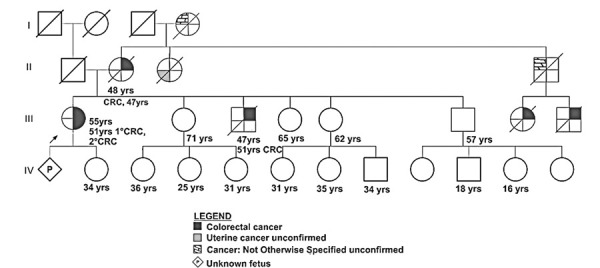
Proband pedigree showing familial segregation of tumors, age of onset, and cancer types. Age in years refers to the current age of the person or age at death. A well-defined age at diagnosis was obtained for III.1 (51-year-old), II.2 (47-year-old), and III.3 (46-year-old)

Family history is relevant to colorectal cancer. The proband’s grandmother had gynecological/uterine cancer. Unfortunately, the proband was unable to provide additional details on family history or add confirmatory reports regarding the type of tumor for distant family members. The family came from Teofilo Otoni, a city in northern Minas Gerais State in southeastern Brazil. Family history met the Bethesda criteria for LS. Definitive evidence for the Amsterdam II criteria was not obtained because all other affected relatives were already deceased, and we did not have access to their histological records ( Figure 1 ). Nonetheless, LS diagnosis was supported by immunohistochemical analysis of the proband’s tumors, evidencing the loss of expression of *MSH2* in one tumor and MSH2 and MSH6 in the other.

After pre-test genetic counseling, mutation screening was conducted in the peripheral blood using a next-generation sequencing (NGS)-based multi-gene panel comprising all MMR genes associated with LS, including *EPCAM.* Mutation screening was performed in a CLIA-approved laboratory (on April 2, 2018). No copy number variation analyses were performed. A novel germline variant was identified, NM_000251.3(MSH2):c.1894_1898del (p.Ile633Lysfs*9), which was heterozygous. In this study, we describe the analysis of the functional impact and potential pathogenicity of this variant by adopting a multistep approach to investigate the possible effects, at the RNA and protein levels.

This bioinformatics analysis included the following steps: 1) Previous descriptions of this variant were searched in ClinVar,^( 19 )^ Database of Genomic Variants,^( 20 )^ Ensembl,^( 21 )^ OMIM, PubMed,^( 22 )^ European Variation Archive,^( 23 )^ GeneCard,^( 24 )^ InSIGHT DNA Variant Database,^( 14 )^ VarSome, and ABraOM;^( 25 )^ 2) Variant impacts were predicted using the Variant Effect Predictor (VEP);^( 21 )^ 3) CDS sequence containing NM_000251.3(MSH2):c.1894_1898del was produced by editing the FASTA format sequence of the *MSH2* (NM_000251.3) CDS, downloaded from the National Center for Biotechnology Information (NCBI);^( 26 )^ 4) This RNA sequence was translated to generate the protein sequence containing NM_000251.3(MSH2):c.1894_1898del (p.Ile633Lysfs*9) using ExPASy Translate;^( 27 )^ 5) MSH2 protein isoforms were searched in the UNIPROT database.^( 28 )^ Initially, two MSH2 protein isoforms were identified (P43246-1 and P43246-2) and compared using Basic Local Alignment Search Tool proteins (BLASTP).^( 29 )^ Isoforms were also predicted using the Variant Effect Predictor (VEP) and GTEx;^( 30 )^ 6) Nonsense-mediated decay (NMD) was evaluated using Mutation Taster 2021;^( 31 )^ 7) Evidence for exon skipping was searched in CDS and protein databases as well as in the literature; 8) Information on MSH2 protein domains was obtained using Pfam;^( 32 )^ 9) Impact of this variant on the MSH2 3D-structure was evaluated by homology modeling using Modeller 9.24,^( 33 )^ with eight models (2O8B, 2O8C, 2O8D, 2O8F, 3THW, 3THX, 3THY, and 3THZ) obtained from PDB.^( 34 )^ Ten models of NM_000251.3(MSH2):c.1894_1898del (p.Ile633Lysfs*9) were generated. Among them, the five models presenting the lowest Discrete Optimizer Protein Energy (DOPE) values were selected to produce Ramachandran plots in PDBsum^( 35 )^ and estimate Z-scores using the PROSA-Web^( 36 )^ (Protein Structure Analyses) software. Best models were selected based on higher PROCHECK/Z-score. Once validated, the model with the highest PROCHECK/Z-score was selected for comparison of the 3D-structures (MSH2 wild-type *versus* NM_000251.3(MSH2):c.1894_1898del (p.Ile633Lysfs*9)) using PyMOL software.^( 37 )^

This project was approved by the Ethics in Research Committee of the *Universidade Federal de Minas Gerais* under the protocols CAAE: 01758418.0.0000.5149; # 3.134.990 and CAAE: 48770621.6.0000.5149; # 4.865.051.

## ❚RESULTS

**Figure 2:**
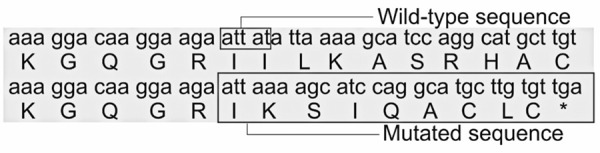
Comparative alignment of MSH2 mRNA and protein, wild-type *vs* . NM_000251.3(MSH2):c.1894_1898del (p.Ile633Lysfs*9) variant. The 5-nucleotide deletion is shown within a box in the wild-type mRNA sequence. Observe that the first codon created after the 5-nucleotide deletion is an ATT and, therefore, the first amino acid in the mutated segment is still an isoleucine (position 632). The sequence changes from codon 633 onwards, to include a stop codon at position 641

NM_000251.3(MSH2):c.1894_1898del (p.Ile633Lysfs*9) was not identified in any of the databases searched. The *MSH2* CDS containing the five-nucleotide deletion induced by this variant and the resulting protein sequence predicted using ExPASy Translate are provided in Table 1S , Supplementary Material. The wild-type MSH2 protein contains 934 amino acids, and this deletion resulted in a reduced protein length to 640 amino acids. Therefore, 294 amino acids at the C-terminus were lost. The five-nucleotide deletion affected codons 632 and 633. After the frameshift mutation (fsm), amino acid 632, an isoleucine residue, did not change. The first change observed in the protein sequence was in amino acid residue 633, which was a substitution of isoleucine with lysine. A premature stop codon was present at the 9th position ( Figure 2 ). We used the NM_000251.3(MSH2):c.1894_1898del (p.Ile633Lysfs*9)-predicted protein sequence to search the NCBI database, using BLASTP. No match for the sequence was identified after the frameshift mutation, supporting the idea that this variant has not been previously reported.

**Table 1S:** *MSH2* CDS and protein sequences predicted in the presence of the NM_000251.3(MSH2):c.1894_1898del (p.Ile633Lysfs*9)

Data S1 MSH2 c.1894_1898del; p.Ile633Lysfs*9
>CDS sequence mutated MSH2 (MSH2 c.1894_1898del; p.Ile633Lysfs*9)
ATGGCGGTGCAGCCGAAGGAGACGCTGCAGTTGGAGAGCGCGGCCGAGGTCGGCTTCGTGCGCTTCTTTC
AGGGCATGCCGGAGAAGCCGACCACCACAGTGCGCCTTTTCGACCGGGGCGACTTCTATACGGCGCACGG
CGAGGACGCGCTGCTGGCCGCCCGGGAGGTGTTCAAGACCCAGGGGGTGATCAAGTACATGGGGCCGGCA
GGAGCAAAGAATCTGCAGAGTGTTGTGCTTAGTAAAATGAATTTTGAATCTTTTGTAAAAGATCTTCTTC
TGGTTCGTCAGTATAGAGTTGAAGTTTATAAGAATAGAGCTGGAAATAAGGCATCCAAGGAGAATGATTG
GTATTTGGCATATAAGGCTTCTCCTGGCAATCTCTCTCAGTTTGAAGACATTCTCTTTGGTAACAATGAT
ATGTCAGCTTCCATTGGTGTTGTGGGTGTTAAAATGTCCGCAGTTGATGGCCAGAGACAGGTTGGAGTTG
GGTATGTGGATTCCATACAGAGGAAACTAGGACTGTGTGAATTCCCTGATAATGATCAGTTCTCCAATCT
TGAGGCTCTCCTCATCCAGATTGGACCAAAGGAATGTGTTTTACCCGGAGGAGAGACTGCTGGAGACATG
GGGAAACTGAGACAGATAATTCAAAGAGGAGGAATTCTGATCACAGAAAGAAAAAAAGCTGACTTTTCCA
CAAAAGACATTTATCAGGACCTCAACCGGTTGTTGAAAGGCAAAAAGGGAGAGCAGATGAATAGTGCTGT
ATTGCCAGAAATGGAGAATCAGGTTGCAGTTTCATCACTGTCTGCGGTAATCAAGTTTTTAGAACTCTTA
TCAGATGATTCCAACTTTGGACAGTTTGAACTGACTACTTTTGACTTCAGCCAGTATATGAAATTGGATA
TTGCAGCAGTCAGAGCCCTTAACCTTTTTCAGGGTTCTGTTGAAGATACCACTGGCTCTCAGTCTCTGGC
TGCCTTGCTGAATAAGTGTAAAACCCCTCAAGGACAAAGACTTGTTAACCAGTGGATTAAGCAGCCTCTC
ATGGATAAGAACAGAATAGAGGAGAGATTGAATTTAGTGGAAGCTTTTGTAGAAGATGCAGAATTGAGGC
AGACTTTACAAGAAGATTTACTTCGTCGATTCCCAGATCTTAACCGACTTGCCAAGAAGTTTCAAAGACA
AGCAGCAAACTTACAAGATTGTTACCGACTCTATCAGGGTATAAATCAACTACCTAATGTTATACAGGCT
CTGGAAAAACATGAAGGAAAACACCAGAAATTATTGTTGGCAGTTTTTGTGACTCCTCTTACTGATCTTC
GTTCTGACTTCTCCAAGTTTCAGGAAATGATAGAAACAACTTTAGATATGGATCAGGTGGAAAACCATGA
ATTCCTTGTAAAACCTTCATTTGATCCTAATCTCAGTGAATTAAGAGAAATAATGAATGACTTGGAAAAG
AAGATGCAGTCAACATTAATAAGTGCAGCCAGAGATCTTGGCTTGGACCCTGGCAAACAGATTAAACTGG
ATTCCAGTGCACAGTTTGGATATTACTTTCGTGTAACCTGTAAGGAAGAAAAAGTCCTTCGTAACAATAA
AAACTTTAGTACTGTAGATATCCAGAAGAATGGTGTTAAATTTACCAACAGCAAATTGACTTCTTTAAAT
GAAGAGTATACCAAAAATAAAACAGAATATGAAGAAGCCCAGGATGCCATTGTTAAAGAAATTGTCAATA
TTTCTTCAGGCTATGTAGAACCAATGCAGACACTCAATGATGTGTTAGCTCAGCTAGATGCTGTTGTCAG
CTTTGCTCACGTGTCAAATGGAGCACCTGTTCCATATGTACGACCAGCCATTTTGGAGAAAGGACAAGGA
AGA-----ATTAAAAGCATCCAGGCATGCTTGTGTTGAAGTTCAAGATGAAATTGCATTTATTCCTAATG
ACGTATACTTTGAAAAAGATAAACAGATGTTCCACATCATTACTGGCCCCAATATGGGAGGTAAATCAAC
ATATATTCGACAAACTGGGGTGATAGTACTCATGGCCCAAATTGGGTGTTTTGTGCCATGTGAGTCAGCA
GAAGTGTCCATTGTGGACTGCATCTTAGCCCGAGTAGGGGCTGGTGACAGTCAATTGAAAGGAGTCTCCA
CGTTCATGGCTGAAATGTTGGAAACTGCTTCTATCCTCAGGTCTGCAACCAAAGATTCATTAATAATCAT
AGATGAATTGGGAAGAGGAACTTCTACCTACGATGGATTTGGGTTAGCATGGGCTATATCAGAATACATT
GCAACAAAGATTGGTGCTTTTTGCATGTTTGCAACCCATTTTCATGAACTTACTGCCTTGGCCAATCAGA
TACCAACTGTTAATAATCTACATGTCACAGCACTCACCACTGAAGAGACCTTAACTATGCTTTATCAGGT
GAAGAAAGGTGTCTGTGATCAAAGTTTTGGGATTCATGTTGCAGAGCTTGCTAATTTCCCTAAGCATGTA
ATAGAGTGTGCTAAACAGAAAGCCCTGGAACTTGAGGAGTTTCAGTATATTGGAGAATCGCAAGGATATG
ATATCATGGAACCAGCAGCAAAGAAGTGCTATCTGGAAAGAGAGCAAGGTGAAAAAATTATTCAGGAGTT
CCTGTCCAAGGTGAAACAAATGCCCTTTACTGAAATGTCAGAAGAAAACATCACAATAAAGTTAAAACAG
CTAAAAGCTGAAGTAATAGCAAAGAATAATAGCTTTGTAAATGAAATCATTTCACGAATAAAAGTTACTA
CGTGA
>Protein sequence mutated of the DNA mismatch repair protein MSH2(MSH2 c.1894_1898del; p.Ile633Lysfs*9).
MAVQPKETLQLESAAEVGFVRFFQGMPEKPTTTVRLFDRGDFYTAHGEDA
LLAAREVFKTQGVIKYMGPAGAKNLQSVVLSKMNFESFVKDLLLVRQYRV
EVYKNRAGNKASKENDWYLAYKASPGNLSQFEDILFGNNDMSASIGVVGV
KMSAVDGQRQVGVGYVDSIQRKLGLCEFPDNDQFSNLEALLIQIGPKECV
LPGGETAGDMGKLRQIIQRGGILITERKKADFSTKDIYQDLNRLLKGKKG
EQMNSAVLPEMENQVAVSSLSAVIKFLELLSDDSNFGQFELTTFDFSQYM
KLDIAAVRALNLFQGSVEDTTGSQSLAALLNKCKTPQGQRLVNQWIKQPL
MDKNRIEERLNLVEAFVEDAELRQTLQEDLLRRFPDLNRLAKKFQRQAAN
LQDCYRLYQGINQLPNVIQALEKHEGKHQKLLLAVFVTPLTDLRSDFSKF
QEMIETTLDMDQVENHEFLVKPSFDPNLSELREIMNDLEKKMQSTLISAA
RDLGLDPGKQIKLDSSAQFGYYFRVTCKEEKVLRNNKNFSTVDIQKNGVK
FTNSKLTSLNEEYTKNKTEYEEAQDAIVKEIVNISSGYVEPMQTLNDVLA
QLDAVVSFAHVSNGAPVPYVRPAILEKGQGRIKSIQACLC

Note: The first sequence denotes a mutated CDS from the *MSH2* gene. The five dashes (-----) denote the 5-nucleotide deletion in position 1894-1898. The second sequence refers to the 640 amino acids, mutated MSH2 protein. The 5-nucleotide deletion produces a frame-shift mutation. Consequently, the protein sequence changes from amino acid residue 633 on and a stop codon is inserted after residue 640.

**Figure 3:**
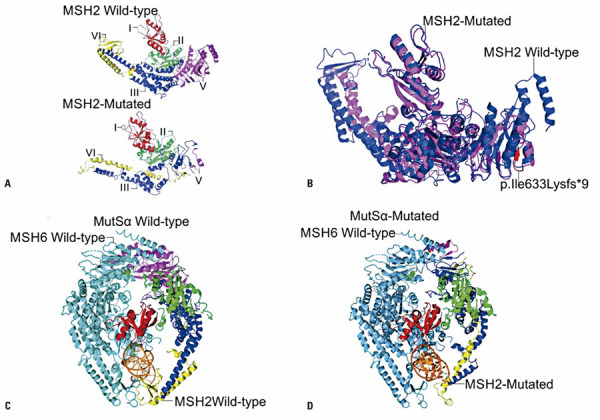
A) Comparison between MSH2 wild-type and NM_000251.3(MSH2):c.1894_1898del p.Ile633Lysfs*9 proteins. MutS I domain is represented in red; MutS II domains in green; MutS III domain in blue; MutS IV domain in yellow; and, MutS V domain in magenta; B) Overlapping blue wild-type MSH2 protein and magenta mutated MSH2 protein. The portion of the protein that is affected by the frame-shift mutation is indicated in red; C) Wild-type MutS alpha complex. Blue contains wild-type MSH2 protein and orange contains wild-type MSH6 protein; D) Mutated MutS alpha complex. Blue shows the mutated MSH2 protein and orange, the wild-type MSH6 protein

Next, we evaluated the effect of NM_000251.3(MSH2):c.1894_1898del (p.Ile633Lysfs*9) on MSH2 protein domains. MSH2 contains 33 protein domains and characteristic molecular features ( Table 2S , Supplementary Material). This variant disrupts exon 12 and leads to the deletion of exons 13 to 16. NM_000251.3(MSH2):c.1894_1898del (p.Ile633Lysfs*9) is located in the loop between MutS domains III/IV and V, causing the loss of domain V, a highly conserved ATPase domain that provides energy for the first steps of MMR.^( 38 )^ The 3D-model of the wild-type MSH2 protein selected using Modeller was 2O8B. Visual inspection of the comparative 3D-models of the wild-type and mutated proteins suggested that this variant affects MSH2 and MSH6 protein interactions ( Figure 3 ).

**Table 2S:** MSH2 isoforms, according to different databases, respective annotation status, and impact of NM_000251.3(MSH2):c.1894_1898del (p.Ile633Lysfs*9) variant

Ensembl isoform name	Transcript ID	bp	Protein	Biotype	Uniprot	Description
MSH2-201	ENST00000233146.7	3115	934 aa	Protein coding	P43246-1	Reviewed (Swiss-Prot): Manually annotated - Peptide RefSeq
MSH2-204	ENST00000543555.6	3018	868 aa	Protein coding	P43246-2	The sequence of this isoform differs from the peptide RefSeq by present alternative splicing, with 66 the loss
MSH2-208	ENST00000645506.1	7893	924 aa	Protein coding	A0A2R8Y6P0	Unreviewed (TrEMBL):Computationally analyzed - Records that await full manual annotation
MSH2-202	ENST00000406134.5	3628	921 aa	Protein coding	E9PHA6	Unreviewed (TrEMBL): Computationally analyzed - Records that await full manual annotation
MSH2-205	ENST00000644092.1	5343	558aa	Nonsense mediated decay	A0A2R8Y7S8	Unreviewed (TrEMBL): Computationally analyzed - Records that await full manual annotation
MSH2-209	ENST00000646415.1	4523	938aa	Nonsense mediated decay	A0A2R8YG02	Unreviewed (TrEMBL): Computationally analyzed - Records that await full manual annotation.
MSH2-207	ENST00000645339.1	3113	918 aa	Nonsense mediated decay	A0A2R8YFH0	Unreviewed (TrEMBL): Computationally analyzed - Records that await full manual annotation
MSH2-206	ENST00000644900.1	1663	168 aa	Nonsense mediated decay	A0A2R8Y713	Unreviewed (TrEMBL): Computationally analyzed - Records that await full manual annotation
MSH2-203	ENST00000467323.1	313	No protein	Processed transcript	-	-
MSH2-003	ENST00000454849.1	560	140 aa	Protein coding	C9J809	This entry is obsolete (isoform excluded from the Ensembl database.)
MSH2-005	ENST00000461394.1	416	No protein	Processed transcript	-	-

Source: adapted from Hunt SE, McLaren W, Gil L, Thormann A, Schuilenburg H, Sheppard D, et al. Ensembl variation resources. Database (Oxford). 2018;2018:bay119.^(21)^

In addition, we evaluated the impact of this variant on different *MSH2* RNA isoforms and overlapping genes and investigated the mechanisms that may reduce the impact of the variant, such as alternative splicing, exon skipping, and NMD. *MSH2* is located in the 2p21-p16.3 region, CHR2:47403067-47634501 (GRCh38 p.13). Typically, *MSH2* is composed of 16 exons; however, this gene contains 46 possible exons. *MSH2* mRNA undergoes alternative splicing. One difficulty in predicting the impact of the *MSH2* variant is that the number of CDS and protein isoforms, as well as their identification numbers, vary according to the source consulted. For example, nine isoforms have been described in Ensembl, and 10 isoforms have been described in GTEx. The CDS and MSH2 protein isoforms linking different nomenclatures are shown in Table 1S . The two canonical MSH2 protein isoforms (P43246-1 and P43246-2) were similarly affected by the NM_000251.3(MSH2):c.1894_1898del (p.Ile633Lysfs*9) variant. P43246-1 was 66 amino acids longer than P43246-2. This difference is due to alternative splicing of the first exon, which encodes a flexible loop at the N-terminus of the protein and does not change the MutS domains. Therefore, the 294 amino acid deletion induced by NM_000251.3(MSH2):c.1894_1898del (p.Ile633Lysfs*9) starts at residue 640 in P43246-1 and residue 576 in the P43246-2 isoform. However, the sequences and, consequently, the variant effects of both isoforms were the same in this region. Additional information on the impact of this variant on different isoforms is provided in Table 1S , Supplementary Material.

There are two additional genes in this region, *MSH2-OT1* and *KCNK2* . *MSH2-OT1* and *KCNK12* overlap with the long *MSH2* isoforms. None of these neighboring genes/isoforms were affected by the NM_000251.3(MSH2):c.1894_1898del (p.Ile633Lysfs*9) variant.

Alternative splicing, which can rescue a mutated allele by generating a shorter but functional protein, has also been investigated.^( 39 )^ No MSH2 protein isoform without exon 12 but preserving NH_2_- and COOH-ends was supported (GTex, accessed May 29, 2025). To date, no such isoform has been reported in either the RNA or protein databases. The predicted impact of NM_000251.3(MSH2):c.1894_1898del (p.Ile633Lysfs*9) on different *MSH2* RNA and protein isoforms is presented in Table 2S , Supplementary Material. Considering the immunohistochemical findings, showing loss of MSH2 protein expression in one tumor and that of MSH2 and MSH6 in the other, we investigated the hypothesis of NMD. The premature termination codon inserted as a consequence of the frameshift mutation occurs in the 12th out of 16 exons, before the last exon-exon boundary (NM_000251.3). Consequently, Mutation Taster predicts that this variant leads to NMD.

## DISCUSSION

In the present study, a patient presenting the LS phenotype with a newly identified germline variant was investigated clinically, immunohistochemically, and at the molecular level. NM_000251.3(MSH2):c.1894_1898del (p.Ile633Lysfs*9) was not found in the aforementioned variant databases or in studies describing Brazilian samples published to date.^( 40 , 41 )^ In VarSome, a similar fsm in the same region, MSH2(ENST00000233146.7):c.1899_1900del p.(Leu634LysfsTer9), is listed as pathogenic; however, the associated phenotypes and molecular mechanisms are not provided. Here, we discuss two aspects: the complexity of establishing a diagnosis of LS in small families and the difficulty of classifying germline variants in relation to their pathogenicity.

First, one of the dilemmas in assisting people with mutations that predispose them to an inherited disease but do not necessarily determine the disease, is how to guide them appropriately through the decision-making process. The main questions concern medical support and balancing the risks and benefits of the available risk-reducing procedures. Some risk-reducing procedures are highly invasive, and the decision-making process requires a solid scientific basis as well as common sense. Since LS is a heterogeneous disease, optimized management may be limited, depending on how well documented and complete the family data is, in order to apply current guidelines.^( 42 )^ Complete family data is frequently difficult to obtain, particularly the actual number of affected relatives, information on the type of neoplasia, age of onset, and tumor documentation. In addition, families tend to be small, and patients may have restrictions on contacting family members; thus, improving the quality of the data may be difficult. Second, we consider the evidence for *MSH2* loss of function and NM_000251.3(MSH2):c.1894_1898del (p.Ile633Lysfs*9) contribution to LS in this family. Loss of *MSH2* expression was implicated by immunohistochemistry, in the tumors of the proband. Identification of NM_000251.3(MSH2):c.1894_1898del (p.Ile633Lysfs*9) confirmed the diagnosis of LS. Frame-shift mutations are automatically classified as pathogenic by most software used in variant pathogenicity classification, and most fsms are probably or certainly pathogenic. However, compensatory cellular mechanisms function at both the RNA and protein levels. At the RNA level, damage-reducing mechanisms include NMD, alternative splicing, and exon skipping.^( 43 , 44 )^ At the protein level, some mechanisms are damaging, such as dominant-negative effects, a phenomenon that occurs in dimeric or multimeric proteins in which the entry of a mutated subunit into the complex induces its precipitation.^( 45 )^ Consequently, the mutated allele reduces the function of the normal allele. However, dominant-negative effects are not observed when the variant affects the protein surface, which interacts with other proteins and prevents it from entering the complexes.^( 46 )^ Therefore, the impact of fsms should be carefully evaluated.

At the RNA level, we searched for evidence of alternative splicing, exon skipping, or NMD. Isoforms described in *MSH2* in both healthy and colon cancer cell lines include the deletion of some exons;^( 47 )^ however, no evidence of alternative splicing or exon skipping resulting in an isoform without exon 12 was found in literature or any database. This variant fulfills the typical requirements for NMD, that is, the creation of a stop codon before the last exon-exon boundary, and consequently, distant from the typical termination of translation.^( 48 )^ The loss of *MSH2* expression observed by immunohistochemistry provides additional evidence of NMD. However, another possible loss-of-function mechanism has emerged. Protein variants causing loss of the C-terminus are frequently driven to degradation, a mechanism that may also explain the loss of MSH2 expression in the proband tumor, as detected by immunohistochemistry.^( 49 )^

Despite the evidence for NMD or protein degradation due to the lack of a C-terminus, we investigated the consequences of NM_000251.3(MSH2):c.1894_1898del (p.Ile633Lysfs*9) at the protein level. In presence of this mutation, MSH2 protein loses 294 C-terminal amino acid residues. NM_000251.3(MSH2):c.1894_1898del (p.Ile633Lysfs*9) is expected to cause the loss of MutS domain V and, consequently, the loss of the interaction with ADP/ATP and the ATPase activity needed for mismatch recognition and DNA binding, at the very beginning of the MMR.^( 3 , 41 , 50 )^ MSH2 interacts with MSH6 and MSH3 to form MutSalpha and MutSbeta, respectively, via MutS domains I and IV. These domains are also involved in DNA binding. MSH2 MutS domains II and III act as connectors between MutS domains V, I, and IV.^( 3 , 41 , 50 )^ At first sight, it appears that the loss of MutS domain V may not prevent the mutated MSH2 protein monomer from entering the MutSalpha or MutSbeta complexes. An additional description of the interaction mechanisms of the MutS complexes involved in MMR is provided in Table 3S , Supplementary Material. We modeled protein complexes containing MSH2 to understand how they may be affected by the presence of NM_000251.3(MSH2):c.1894_1898del (p.Ile633Lysfs*9) ( Figure 3 ). The results suggest that this variant prevents MSH2 from interacting with MSH6 to form the MutSalpha complex. No evidence of the ability of MSH2 or MSH3 to form MutSbeta was found.

**Table 3S:** Interaction mechanisms of MutS complexes in Mismatch Repair

.
The intera ction with DNA mismatches or insertion/deletion loops occurs initially with the MutS domains I of both subunits (MSH2/MSH36 or MSH23/MSH6) accessing the DNA minor groove. The MutSbeta complex induces the distortion of the sugar-phosphate structure in the DNA segment presenting the mismatch (Gupta, Gellert et al. 2012). The MutSbeta and MutSalpha complexes exchange ADP for ATP to move the DNA strand and to interact with downstream effectors, such as PCNA, MLH1/PMS2, and EXO1. Once recruited, PCNA is responsible for starting the excision of the newly replicated DNA strand. The MLH1/PMS2 complex has endonucleolytic activity, thus being able to create a cut in the nucleotide segment that needs to be repaired, allowing EXO1 to remove hundreds of nucleotides ( Fukui 2010 ; Martín-López and Fishel 2013 ; Edelbrock, Kaliyaperumal et al. 2009). Consequently, the downstream MMR cascade would be affected by the loss of MutSbeta function. After evaluating the consequences of MSH2 c.1894_1898del; (p.Ile633Lysfs*9) variant, we consider that it fulfills the criteria for pathogenicity accepted for fsm in MSH2 (Plazzer, Sijmons et al. 2013).

After considering the overall results, we found no evidence that NM_000251.3(MSH2):c.1894_1898del (p.Ile633Lysfs*9) is a non-pathogenic variant. The strengths of the present study are as follows. 1) This variant was not found in any of the population databases. It has been included in VarSome, but without additional information; 2) It causes the loss of MutS domain V, and therefore, the ADP binding ability of the MSH2 protein; 3) There is no evidence that the effects of this variant may be ameliorated by physiological alternative splicing or exon skipping. The large number of exon 12 pathogenic variants in InSIGHT suggests that no such rescue mechanisms are possible; 4) There is evidence of NMD; and, 5). In case this mRNA escapes NMD, the resulting protein may possibly enter MutSalpha complexes; however, these complexes would be less compact than those established by the normal allele and probably non-functional because of the loss of the MSH2 ADP binding site. Regarding limitations of the study *,* no *in vitro* analyses were performed to validate or exclude the mechanisms identified *in silico* . However, according to the ACMG criteria, *in vitro* or *in vivo* analyses are not formally required when loss of function is a highly probable consequence of a genetic variant (e.g., an fsm). NM_000251.3(MSH2):c.1894_1898del (p.Ile633Lysfs*9) fulfills the criteria for PVS1 (null variant - nonsense, frameshift, canonical +/- 1 or splice sites, initiation codon, and single or multiple exon deletion). It also fulfills the PM2 criterion (absent from controls or present with extremely low frequency, if recessive, in the Exome Sequencing Project,^( 51 )^ 1000 Genomes Project,^( 52 )^ or Exome Aggregation Consortium,^( 53 )^ which supports its classification as pathogenic. The additional bioinformatics analysis described here provided no evidence of rescue mechanisms acting to alleviate the effects of NM_000251.3(MSH2):c.1894_1898del (p.Ile633Lysfs*9). The bioinformatic analysis strategy proposed in this study was limited by the absence of validation by analysis of similar variants, in other genes.

## CONCLUSION

In the present study, we used several different bioinformatics tools to evaluate the NM_000251.3(MSH2):c.1894_1898del (p.Ile633Lysfs*9) germline variant identified in a patient with Lynch syndrome. No evidence was identified for a compensatory mechanism that may reduce the impact of NM_000251.3(MSH2):c.1894_1898del (p.Ile633Lysfs*9). In conclusion, the evidence reported in this study indicates that NM_000251.3(MSH2):c.1894_1898del p.Ile633Lysfs*9 is a likely pathogenic variant that can explain the occurrence of Lynch syndrome in this family.

## DATA AVAILABILITY:

Data are available to reviewers upon request.
